# Ultra-narrow metallic armchair graphene nanoribbons

**DOI:** 10.1038/ncomms10177

**Published:** 2015-12-14

**Authors:** Amina Kimouche, Mikko M. Ervasti, Robert Drost, Simo Halonen, Ari Harju, Pekka M. Joensuu, Jani Sainio, Peter Liljeroth

**Affiliations:** 1 Department of Applied Physics, Aalto University School of Science, PO Box 15100, Aalto 00076, Finland; 2 COMP Centre of Excellence, Department of Applied Physics, Aalto University School of Science, PO Box 11100, Aalto 00076, Finland; 3 Department of Chemistry, Aalto University School of Chemical Technology, PO Box 16100, Aalto 00076, Finland

## Abstract

Graphene nanoribbons (GNRs)—narrow stripes of graphene—have emerged as promising building blocks for nanoelectronic devices. Recent advances in bottom-up synthesis have allowed production of atomically well-defined armchair GNRs with different widths and doping. While all experimentally studied GNRs have exhibited wide bandgaps, theory predicts that every third armchair GNR (widths of *N*=3*m*+2, where *m* is an integer) should be nearly metallic with a very small bandgap. Here, we synthesize the narrowest possible GNR belonging to this family (five carbon atoms wide, *N*=5). We study the evolution of the electronic bandgap and orbital structure of GNR segments as a function of their length using low-temperature scanning tunnelling microscopy and density-functional theory calculations. Already GNRs with lengths of 5 nm reach almost metallic behaviour with ∼100 meV bandgap. Finally, we show that defects (kinks) in the GNRs do not strongly modify their electronic structure.

## Abstract (web summary)

Graphene nanoribbons have potential applications as nanoscale wires, though experimentally studied ribbons display wide bandgaps. Here, the authors synthesise the narrowest armchair graphene nanoribbon predicted to have metallic behaviour and show these ribbons—5 carbon atoms wide—indeed display almost metallic behaviour.

## Graphical abstract

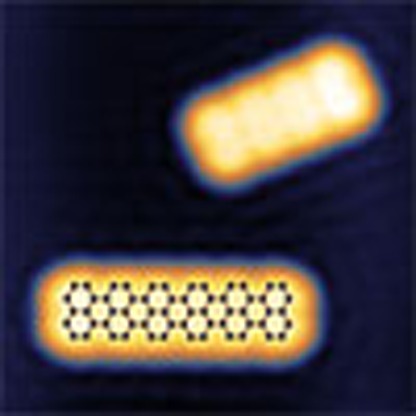

Graphene nanoribbons (GNRs) are a new class of materials that have promising applications in next-generation nanoelectronic and optoelectronic devices^1^,^2^,^3^. These systems have been thoroughly studied theoretically at various levels of sophistication^4^,^5^,^6^,^7^,^8^,^9^. According to these studies, the electronic and magnetic properties can be tuned by the nanoribbon chemical structure and edge geometry, ranging from semiconductors^7^,^8^ to spin-polarized half-metals^6^. Zigzag edge structure is predicted to result in spin-polarized edge states, with potential applications in spintronics^4^,^6^,^10^. On the other hand, in armchair GNRs, quantum confinement opens a bandgap that sensitively depends on the ribbon width, allowing in principle for the design of GNR-based structures with tuneable properties. The armchair ribbons can be grouped into three families, that is, *N*=3*p*, *N*=3*p+1* and *N*=3*p*+2, where *p* is an integer and *N* the number of carbon atoms along the GNR width^4^,^11^. *N*=3*p* and *N*=3*p*+1 families have wide bandgaps that scale inversely with the ribbon width. In contrast, simple models predict the family *N*=3*p*+2 to be metallic with zero bandgap^4^,^12^. More sophisticated models taking into account, for example, edge relaxation predict non-vanishing but small bandgaps^8^,^13^. These narrow armchair GNRs with narrow or vanishing bandgaps would form ideal molecular wires to be used as interconnects in molecular scale circuitry. However, this requires atomic-level control of the edge structure, which is far beyond the existing top–down approaches such as electron beam lithography. Recently, tremendous progress has been made in bottom-up chemical synthesis of GNRs from molecular precursors^14^,^15^,^16^,^17^,^18^,^19^,^20^,^21^,^22^,^23^. The synthesis of these ribbons proceeds in two thermally activated steps^14^. First step is the cleavage of halogens from the precursors and the formation of a covalently coupled polymer through radical addition reaction. The linear chain is converted into graphene in the second step that involves cyclodehydrogenation at a higher temperature. By changing the monomer design, the fabrication of a wide range of GNRs including different widths and doping can be achieved.

Despite several kinds of armchair GNRs having been synthesized via specific molecular precursors^2^,^16^,^17^,^18^,^19^,^21^,^22^,^23^,^24^,^25^,^26^, the studied widths have been rather limited. Most of them belong to the wide bandgap *N*=3*p*+1 family and narrow bandgap, near-metallic behaviour has not been verified experimentally. We focus on the *N*=3*p*+2 family and target the narrowest member of this family with *N*=5. The synthesis starts with a dibromoperylene molecule, which undergoes dehalogenation and cyclodehydrogenation steps to yield atomically perfect *N*=5 armchair GNRs. This width is predicted to be metallic within a nearest-neighbour tight-binding model^4^,^7^. More realistic calculations predict the presence of a bandgap, but it should remain much smaller than that found in armchair GNRs of the other families^7^. We confirm these theoretical predictions and measure experimentally a ∼100 meV bandgap in long GNRs using low-temperature scanning tunnelling microscopy (STM). This near-metallic regime is already reached in GNRs of six perylene monomer units, that is, with lengths longer than 5 nm. In addition, we study the effect of the ribbon length on the electronic structure and map out the local density of states (LDOS) in real space using scanning tunnelling spectroscopy. The experimental results are corroborated by density-functional theory (DFT) calculations that are used to identify the fingerprints of molecular orbitals as a function of the GNR length. Finally, we show that defects (kinks) in the GNRs do not destroy the near-metallic behaviour. Our results demonstrate that this armchair GNR subfamily can be used as molecular wires that should exhibit metallic behaviour at room temperature. This suggest that these GNRs would form ideal interconnects in molecular scale electronic circuitry.

## Results

### Growth of *N*=5 armchair GNRs

**Figure 1:**
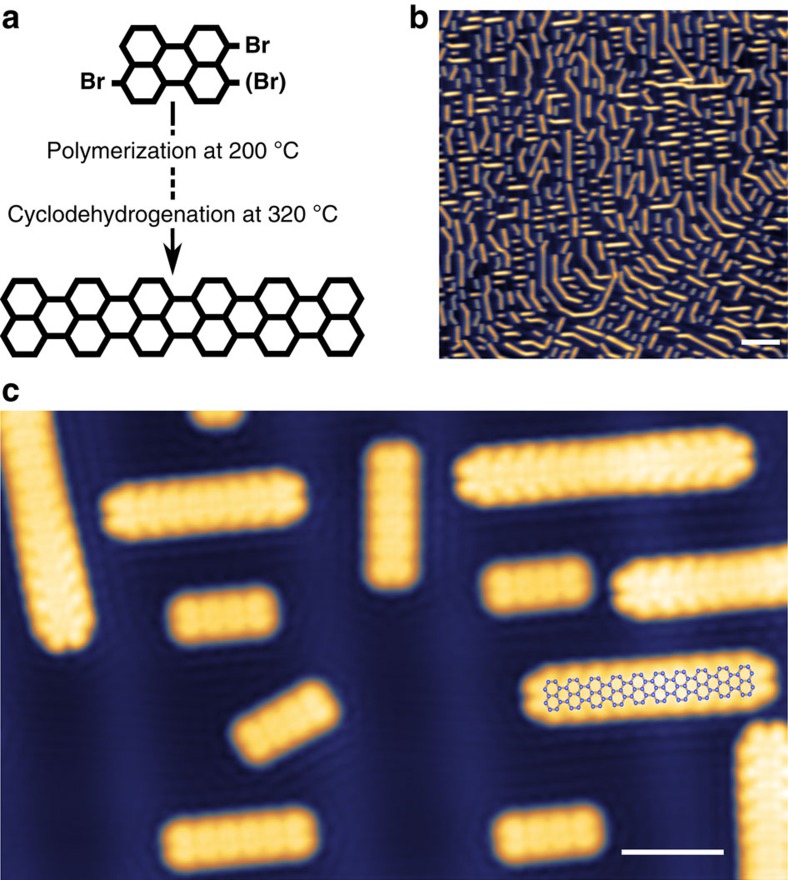
Bottom-up synthesis of *N*=5 armchair GNRs. (**a**) Reaction scheme of the polymerization of the DBP precursor to atomically well defined *N*=5 armchair GNRs. (**b**) Overview STM image after cyclodehydrogenation at 320 °C, showing straight and kinked GNRs (*V*=500 mV, *I*=50 pA; scale bar, 10 nm). (**c**) Zoomed-in STM topography of different ribbon lengths (*V*=300 mV, *I*=50 pA; scale bar, 2 nm).

We grew armchair GNRs in ultrahigh vacuum (UHV) using the on-surface polymerization with dibromoperylene C_20_H_10_Br_2_ (DBP) as the molecular precursor (Fig. 1a)^23^. This process yields very narrow 5-GNRs with armchair edge structure. Details of the sample preparation can be found in the Methods section. The resulting GNRs were characterized by low-temperature STM at *T*=5 K. We used a mixture of precursor monomers with either parallel or antiparallel positions of Br atoms (3,9-DBP or 3,10-DBP). Regardless of the positions of the Br atoms, fully conjugated ribbons are formed. A typical overview STM scan is shown in Fig. 1b. These ribbons align and assemble along or perpendicular to the direction of the herringbone reconstruction of the Au(111) substrate. Most ribbons are isolated and easily manipulated along the ribbon axis due to the weak interaction with the underlying Au(111) substrate. While most of the GNRs are straight, there is a significant number of non-straight GNRs. These kinked ribbons are formed by two (or more) straight segments connecting during the initial polymerization step with an angle of 30°. The structure and electronic properties of these kinked ribbons will be discussed in detail later. Statistics on the ribbon lengths and the fraction of kinked ribbons are given in Supplementary Fig. 1. Figure 1c shows a high-resolution STM image with GNRs with several different lengths. The shortest ribbons we observe consist of two monomers, a medium length ribbon of 5-monomer units is highlighted with an overlaid model structure. We can easily determine the number of monomer units by counting the number of benzene rings (lobes) along the armchair edges of the GNRs.

### Evolution of GNR orbitals as a function of length

**Figure 2:**
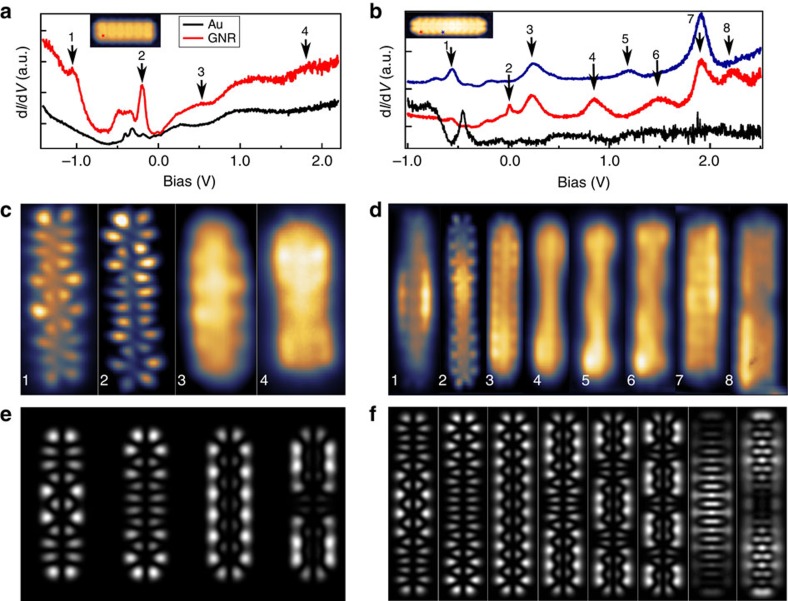
d*I*/d*V* spectroscopy and real-space imaging of the GNR wavefunctions. (**a**,**b**) d*I*/d*V* spectra acquired on three (**a**) and five (**b**) monomer GNR with a metallic tip. Location of the spectra are marked in the STM topographies in the insets and the black curve is measured on Au(111). The red and blue curves are shifted for clarity. (**c-f**) Experimental constant-height d*I*/d*V* maps (**c**,**d**) and the corresponding calculated LDOS maps (**e**,**f**) for three (**c**,**e**) and five (**d**,**f**) monomer GNRs at bias voltages corresponding to the positions marked with arrows in **a**,**b**.

We have performed detailed differential conductance (d*I*/d*V*) spectroscopy experiments as a function of the ribbon length to probe the corresponding changes in their electronic structure. In the following, we will use two ribbon lengths (3 and 5 monomer units) to highlight how the ribbon electronic states change with length. A characteristic spectrum (red curve, Fig. 2a) recorded close to the edge of a 3-monomer GNR exhibits a pronounced shoulder at −1.02 V and a prominent peak at −160 mV (labelled states 1 and 2, respectively). These states correspond to the highest occupied molecular orbitals HOMO-1 (state 1) and HOMO (state 2). At positive bias, we probe the lowest unoccupied states. We find states at 540 mV (state 3) and 1.8 V (state 4), which correspond to the lowest and second lowest unoccupied molecular orbitals (LUMO and LUMO+1). The additional feature observed in the d*I*/d*V* spectrum around 1.2 V did not show any contrast in the d*I*/d*V* map and it originates from the background signal (black line).

We probe the symmetries of the different molecular orbitals by mapping the spatially resolved d*I*/d*V* signal at the energies corresponding to the resonances (Fig. 2c). The experimental maps show that both the HOMO and LUMO are extended through the ribbon, in contrast to the localized end states observed on wider armchair GNRs^2^,^19^. The LUMO+1 has one more nodal plane compared with the LUMO as expected. The corresponding calculated LDOS maps (Fig. 2e) are in excellent agreement with the experimental results, clearly reproducing the occupied and unoccupied states. The unoccupied orbitals yield broader resonances in the d*I*/d*V* spectra and their spatial maps are not as well resolved as for the occupied states. This is likely to be caused by shorter lifetime of the tunnelling electrons on these states leading to increased lifetime broadening. Nevertheless, the orbitals can be identified by the conductance mapping.

Figure 2b shows the d*I*/d*V* spectra acquired at different locations along a 5-monomer GNR. The corresponding experimental and calculated conductance maps are shown in Fig. 2d,f. The occupied states are found at −550 and 26 mV referred as HOMO-1 and HOMO, respectively. The orbital corresponding to the HOMO of an isolated ribbon is found at positive bias, that is, the ribbon has become positively charged on the Au(111) substrate. We find this transition to occur between 4- and 5-monomer long ribbons. It is consistent with the known p-doping of bulk graphene on a gold substrate^27^,^28^. The unoccupied states found at 250 mV, 850 mV, 1.25 V and 1.57 V are referred to as LUMO, LUMO+1, LUMO+2 and LUMO+3, respectively. These unoccupied states follow the energies expected for one-dimensional particle-in-a-box states of massless Dirac fermions. In addition to these resonances corresponding to single molecular orbitals, there are two further peaks (peaks 7 and 8 in Fig. 2b) in the experimental d*I*/d*V* spectra. According to the DFT calculations, these correspond to several closely spaced orbitals (Supplementary Fig. 2). This is further corroborated by simulated LDOS maps that nicely reproduce the spatial features of the experimental maps.

**Figure 3:**
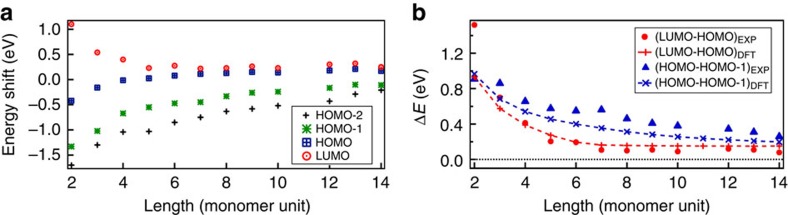
Energies of molecular orbitals as function of ribbon length. (**a**) Energies of the different molecular orbitals (HOMO-2 to LUMO) as a function of the ribbon length. (**b**) Comparison between experimental and calculated energy gaps as a function of the ribbon length.

In addition to quantum confinement, which makes the electronic structure of finite graphene ribbons very sensitive to the width, finite-size effects have to be taken into account to determine the scaling of the HOMO–LUMO energy gap with the length of the ribbon. Here, we will examine how the finite-size effects affect the energies of molecular levels in the transition from short to long ribbons. Experimental orbital energies with respect to Fermi level extracted from d*I*/d*V* spectra are depicted in Fig. 3a for different ribbon lengths. As can be seen, the gap between the highest occupied and the lowest unoccupied molecular orbitals closes quickly with increasing ribbon length. As indicated earlier, the HOMO level crosses the Fermi level between 4- and 5-monomer long ribbons, that is, the ribbon becomes positively charged (this is illustrated in Supplementary Fig. 3). The middle of the gap extrapolates to 0.20±0.02 eV versus gold Fermi level, in agreement with the doping of bulk graphene on Au(111)^27^,^28^. There is no significant abrupt shift of the orbital energies due to the charging of the ribbons, which indicates that the charging energy is small and can be neglected^29^. This also implies that the STM transport gap is nearly equal to the single-particle HOMO–LUMO gap.

We plot the HOMO–LUMO gap as a function of the length of the ribbon in Fig. 3b along with values from the DFT calculations on isolated ribbons. The HOMO–LUMO gap decreases very quickly with increasing ribbon length (scaling roughly as 1/*L*^2^) and is below 200 meV already for 5-monomer ribbons (length of ca. 4 nm). It saturates to value of 0.10±0.02 eV corresponding to a small but finite energy gap in long ribbons. The spacing between the HOMO and HOMO-1 also decreases, but with a different scaling (roughly 1/*L*). This scaling indicates that these states can be viewed as quantum confined levels of particles with linear dispersion as expected in graphene. The HOMO-1–HOMO gap extrapolates to 0 as one would expect for the level spacing within a subband in infinite ribbons. These experimental observations are well reproduced by DFT calculations.

Detailed comparison between theory and experiment shows that DFT overestimates the experimental HOMO–LUMO gap. This is likely to be caused by the omission of the substrate, the resulting charging of the ribbon and the strong metallic screening. Furthermore, the neutral isolated ribbons are predicted to have singlet edge states for lengths >7 monomer units (Supplementary Fig. 4 and Supplementary Methods). The narrow bandgap predicted earlier by DFT^7^ matches very well with our measured and calculated gaps. These values differ from those reported recently by Zhang *et al*.^18^, who obtained a bandgap of 2.8 eV. They used another precursor molecule (tetrabromonaphthalene), which also results in *N*=5 armchair GNRs. Comparison with our results suggests that they have identified the most prominent features in the spectra as the conduction and valence band edges (see for example the peak at around 2.0 V in Fig. 2b). However, our experiments show that there are other, lower lying orbitals and the actual HOMO–LUMO gap on Au(111) reaches value of ∼100 meV in long ribbons. Finally, the many-body phenomena predicted for wider armchair ribbons^30^,^31^ or for zigzag ribbons^32^,^33^,^34^ are not expected for such a short segment of zigzag edge.

### Kinked GNRs formed by connected straight segments

**Figure 4:**
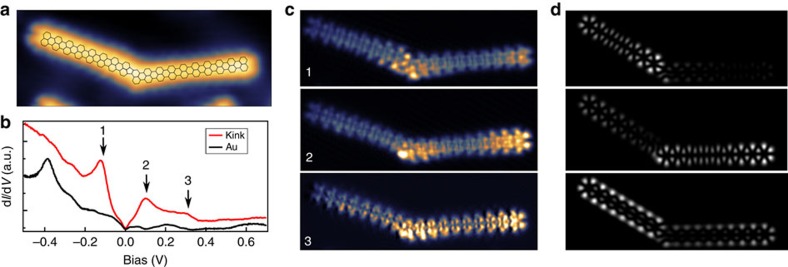
Electronic structure of a kinked GNR with four- and five-monomer segments. (**a**) STM topography with an overlaid model shows the connection on the kink by a pentagon. (**b**) d*I*/d*V* spectrum acquired at the kink (red line), an Au(111) spectrum (black line) is provided for comparison. (**c**) Constant-height d*I*/d*V* maps at energies labelled by arrows in **b**. (**d**) Corresponding simulated LDOS maps.

In addition to straight GNRs, also kinked ribbons are formed (see Fig. 1a). These ribbons typically consist of two straight segments connected at an angle of 150° corresponding to a rotation of 30° (Fig. 4a). Grain boundaries in graphene are known to accommodate carbon pentagon and heptagon defects^35^,^36^. Considering how two nanoribbons might connect, the creation of a pentagon does not require adding or removing carbon atoms from two straight ribbons. In addition, DFT calculations on GNRs with pentagon defects reproduce the experimental results better than structures with heptagons (see below and Supplementary Fig. 5).

We have measured d*I*/d*V* spectra as well conductance maps on kinked ribbons and Fig. 4 shows a ribbon where 4-monomer and 5-monomer long straight segments are joined. Figure 4b shows a d*I*/d*V* spectrum acquired at the kink exhibiting peaks at −120 and 100 mV and a pronounced shoulder at 300 mV. These energies correspond to the HOMO−1, HOMO and LUMO, respectively. Simulated d*I*/d*V* maps for a pentagon connection (HOMO−1, HOMO and LUMO shown in Fig. 4d) match the experimental results reasonably. The energy gap of the kinked ribbon is ca. 200 meV, which is close to a straight 9-monomer ribbon and smaller than expected for four- or five-monomer long GNRs. In addition, the molecular orbitals remain delocalized over the whole ribbon and hence, the kinks would not be expected to strongly affect the GNR conductivity.

## Discussion

Our results demonstrate nearly metallic behaviour in ultra-narrow, atomically well-defined graphene nanoribbons. We have synthesized the narrowest member (*N*=5) of the *N*=3*p*+2 family that has been predicted to exhibit narrow bandgap. We have confirmed this prediction and studied the evolution of the electronic states as a function of the ribbon length in detail using low-temperature STM and numerical methods. A bandgap of ∼100 meV is reached already in short ribbons of only 5 nm in length. In addition, kinked GNRs formed by connected straight armchair GNR segments also exhibit narrow gaps and delocalized electronic orbitals.

These experimental results suggest that this armchair GNR subfamily can be used as molecular wires, which should exhibit metallic behaviour at room temperature. This opens up new opportunities to connect different functional molecules in molecular electronic devices. In addition, it could be possible to integrate these nanoribbons with existing technology, where they have been suggested to outperform the present Cu interconnects both in terms of conductance properties and increased resistance to electromigration^37^,^38^,^39^,^40^,^41^.

## Methods

### Sample preparation

Experiments were conducted under UHV conditions. The substrate was a thin Au(111) film deposited on a (111)-terminated Ir single crystal. The Ir crystal is cleaned by repeated sputtering/annealing cycles at high temperature. Gold (Goodfellow, 99.995%) was deposited from a high-temperature effusion cell at 1,180 °C. The deposition time was 2 h and the sample was kept at 310 °C during the deposition. Growth of GNRs was carried out in a two-step process in which the molecular precursor, dibromoperylene C_20_H_10_Br_2_ (see Supplementary Methods for synthesis information) is first thermally sublimed onto the sample. During the first thermal activation step at 200 °C, the biradical species produced after detachment of bromine diffuse along the surface to form a linear polymer chain. Subsequent annealing of the sample at 320 °C leads to dehydrogenation resulting in the formation of C–C bonds and fully conjugated GNRs with a well-defined width.

After the growth, the sample was inserted into a low-temperature STM operated at 5 K (Unisoku USM-1300), housed within the same UHV system. A Pt–Ir tip was used for topographic and spectroscopic measurements. Scanning tunnelling spectroscopy measurements were performed by lock-in amplifier (frequency=719 Hz, amplitude=15 mV (r.m.s.)) under open feedback.

### DFT calculations

First-principles calculations of *N*=5 armchair GNR were performed using DFT as implemented in the FHI-aims package^42^. The default tight basis sets were used together with the Perdew–Burke–Ernzerhof exchange-correlation functional in a spin-polarized calculation. The structure was fully relaxed until the force on each atom was smaller than 0.001 eV Å^−1^ and total energies were converged to 10^−6^ eV. In the LDOS simulations, a Lorentzian broadening of 0.03 eV of all the orbitals was used. Further details can be found in the Supplementary Information.

## Additional information

**How to cite this article:** Kimouche, A. *et al*. Ultra-narrow metallic armchair graphene nanoribbons. *Nat. Commun.* 6:10177 doi: 10.1038/ncomms10177 (2015).

## Supplementary Material

Supplementary Information Supplementary Figures 1-5, Supplementary Methods and Supplementary References

## References

[b1] Li X. L., Wang X. R., Zhang L., Lee S. W. & Dai H. J. Chemically derived, ultrasmooth graphene nanoribbon semiconductors. Science 319, 1229–1232 (2008). 18218865 10.1126/science.1150878

[b2] Koch M., Ample F., Joachim C. & Grill L. Voltage-dependent conductance of a single graphene nanoribbon. Nat. Nanotechnol. 7, 713–717 (2012). 23064554 10.1038/nnano.2012.169

[b3] Silveiro I., Ortega J. M. P. & de Abajo F. G. Quantum nonlocal effects in individual and interacting graphene nanoribbons. Light. Sci. Appl. 4, e241 (2015).

[b4] Nakada K., Fujita M., Dresselhaus G. & Dresselhaus M. S. Edge state in graphene ribbons: Nanometer size effect and edge shape dependence. Phys. Rev. B 54, 17954–17961 (1996). 10.1103/physrevb.54.17954 9985930

[b5] Wakabayashi K., Fujita M., Ajiki H. & Sigrist M. Electronic and magnetic properties of nanographite ribbons. Phys. Rev. B 59, 8271–8282 (1999).

[b6] Son Y.-W., Cohen M. L. & Louie S. G. Half-metallic graphene nanoribbons. Nature 444, 347–349 (2006). 17108960 10.1038/nature05180

[b7] Son Y.-W., Cohen M. L. & Louie S. G. Energy gaps in graphene nanoribbons. Phys. Rev. Lett. 97, 216803 (2006). 17155765 10.1103/PhysRevLett.97.216803

[b8] Barone V., Hod O. & Scuseria G. E. Electronic structure and stability of semiconducting graphene nanoribbons. Nano Lett. 6, 2748–2754 (2006). 17163699 10.1021/nl0617033

[b9] Yazyev O. V. A guide to the design of electronic properties of graphene nanoribbons. Acc. Chem. Res. 46, 2319–2328 (2013). 23282074 10.1021/ar3001487

[b10] Yazyev O. V. Emergence of magnetism in graphene materials and nanostructures. Rep. Prog. Phys. 73, 056501 (2010).

[b11] Yang L., Park C.-H., Son Y.-W., Cohen M. L. & Louie S. G. Quasiparticle energies and band gaps in graphene nanoribbons. Phys. Rev. Lett. 99, 186801 (2007). 17995426 10.1103/PhysRevLett.99.186801

[b12] Brey L. & Fertig H. A. Electronic states of graphene nanoribbons studied with the Dirac equation. Phys. Rev. B 73, 235411 (2006).

[b13] Ezawa M. Peculiar width dependence of the electronic properties of carbon nanoribbons. Phys. Rev. B 73, 045432 (2006).

[b14] Cai J. . Atomically precise bottom-up fabrication of graphene nanoribbons. Nature 466, 470–473 (2010). 20651687 10.1038/nature09211

[b15] Zhang X. . Experimentally engineering the edge termination of graphene nanoribbons. ACS Nano 7, 198–202 (2013). 23194280 10.1021/nn303730v

[b16] Blankenburg S. . Intraribbon heterojunction formation in ultranarrow graphene nanoribbons. ACS Nano 6, 2020–2025 (2012). 22324827 10.1021/nn203129a

[b17] Bronner C. . Aligning the band gap of graphene nanoribbons by monomer doping. Angew. Chem. Int. Ed. 52, 4422–4425 (2013). 10.1002/anie.201209735 23512734

[b18] Chen Y.-C. . Tuning the band gap of graphene nanoribbons synthesized from molecular precursors. ACS Nano 7, 6123–6128 (2013). 23746141 10.1021/nn401948e

[b19] van der Lit J. . Suppression of electron–vibron coupling in graphene nanoribbons contacted via a single atom. Nat. Commun. 4, 2023 (2013). 23756598 10.1038/ncomms3023

[b20] Cai J. . Graphene nanoribbon heterojunctions. Nat. Nanotechnol. 9, 896–900 (2014). 25194948 10.1038/nnano.2014.184

[b21] Chen Y.-C. . Molecular bandgap engineering of bottom-up synthesized graphene nanoribbon heterojunctions. Nat. Nanotechnol. 10, 156–160 (2015). 25581888 10.1038/nnano.2014.307

[b22] Zhang H. . On-surface synthesis of rylene-type graphene nanoribbons. J. Am. Chem. Soc. 137, 4022–4025 (2015). 25775004 10.1021/ja511995r

[b23] Sakaguchi H. . Width-controlled sub-nanometer graphene nanoribbon films synthesized by radical-polymerized chemical vapor deposition. Adv. Mater. 26, 4134–4138 (2014). 24711068 10.1002/adma.201305034

[b24] Linden S. . Electronic structure of spatially aligned graphene nanoribbons on Au(788). Phys. Rev. Lett. 108, 216801 (2012). 23003288 10.1103/PhysRevLett.108.216801

[b25] Söde H. . Electronic band dispersion of graphene nanoribbons via Fourier-transformed scanning tunneling spectroscopy. Phys. Rev. B 91, 045429 (2015).

[b26] Talirz L. . Termini of bottom-up fabricated graphene nanoribbons. J. Am. Chem. Soc. 135, 2060–2063 (2013). 23350872 10.1021/ja311099k

[b27] Joseph M. W. . Extraordinary epitaxial alignment of graphene islands on Au(111). New J. Phys. 14, 053008 (2012).

[b28] Leicht P. . In situ fabrication of quasi-free-standing epitaxial graphene nanoflakes on gold. ACS Nano 8, 3735–3742 (2014). 24694063 10.1021/nn500396c

[b29] Swart I., Gross L. & Liljeroth P. Single-molecule chemistry and physics explored by low-temperature scanning probe microscopy. Chem. Commun. 47, 9011–9023 (2011). 10.1039/c1cc11404b 21584325

[b30] Ijäs M. . Electronic states in finite graphene nanoribbons: Effect of charging and defects. Phys. Rev. B 88, 075429 (2013).

[b31] Golor M., Koop C., Lang T. C., Wessel S. & Schmidt M. J. Magnetic correlations in short and narrow graphene armchair nanoribbons. Phys. Rev. Lett. 111, 085504 (2013). 24010454 10.1103/PhysRevLett.111.085504

[b32] Rivero P., Jiménez-Hoyos C. A. & Scuseria G. E. Entanglement and polyradical character of polycyclic aromatic hydrocarbons predicted by projected Hartree–Fock theory. J. Phys. Chem. B 117, 12750–12758 (2013). 23668255 10.1021/jp401478v

[b33] Mizukami W., Kurashige Y. & Yanai T. More π electrons make a difference: Emergence of many radicals on graphene nanoribbons studied by ab initio DMRG theory. J. Chem. Theory Comput. 9, 401–407 (2013). 26589042 10.1021/ct3008974

[b34] Wu C.-S. & Chai J.-D. Electronic properties of zigzag graphene nanoribbons studied by TAO-DFT. J. Chem. Theory Comput. 11, 2003–2011 (2015). 10.1021/ct500999m 26894252

[b35] Terrones H. & Mackay A. L. The geometry of hypothetical curved graphite structures. Carbon 30, 1251–1260 (1992).

[b36] Huang P. Y. . Grains and grain boundaries in single-layer graphene atomic patchwork quilts. Nature 469, 389–392 (2011). 21209615 10.1038/nature09718

[b37] Naeemi A. & Meindl J. D. Conductance modeling for graphene nanoribbon (GNR) interconnects. IEEE Electron Device Lett. 28, 428–431 (2007).

[b38] Murali R., Yang Y., Brenner K., Beck T. & Meindl J. D. Breakdown current density of graphene nanoribbons. Appl. Phys. Lett. 94, 243114 (2009).

[b39] Murali R., Brenner K., Yinxiao Y., Beck T. & Meindl J. D. Resistivity of graphene nanoribbon interconnects. IEEE Electron Device Lett. 30, 611–613 (2009).

[b40] Rakheja S., Kumar V. & Naeemi A. Evaluation of the potential performance of graphene nanoribbons as on-chip interconnects. Proc. IEEE 101, 1740–1765 (2013).

[b41] Schwierz F. Graphene transistors. Nat. Nanotechnol. 5, 487–496 (2010). 20512128 10.1038/nnano.2010.89

[b42] Blum V. . Ab initio molecular simulations with numeric atom-centered orbitals. Comp. Phys. Commun. 180, 2175–2196 (2009).

