# Supplementary material for: Ultra-narrow metallic armchair graphene nanoribbons
Source: Nat Commun. 2015 Dec 14;6:10177. doi: 10.1038/ncomms10177 (PMC4682157; doi:10.1038/ncomms10177)
Supplement: Supplementary Information — Supplementary Figures 1-5, Supplementary Methods and Supplementary References [file ncomms10177-s1.pdf]

Supporting Information for Ultra-narrow metallic armchair graphene nanoribbons

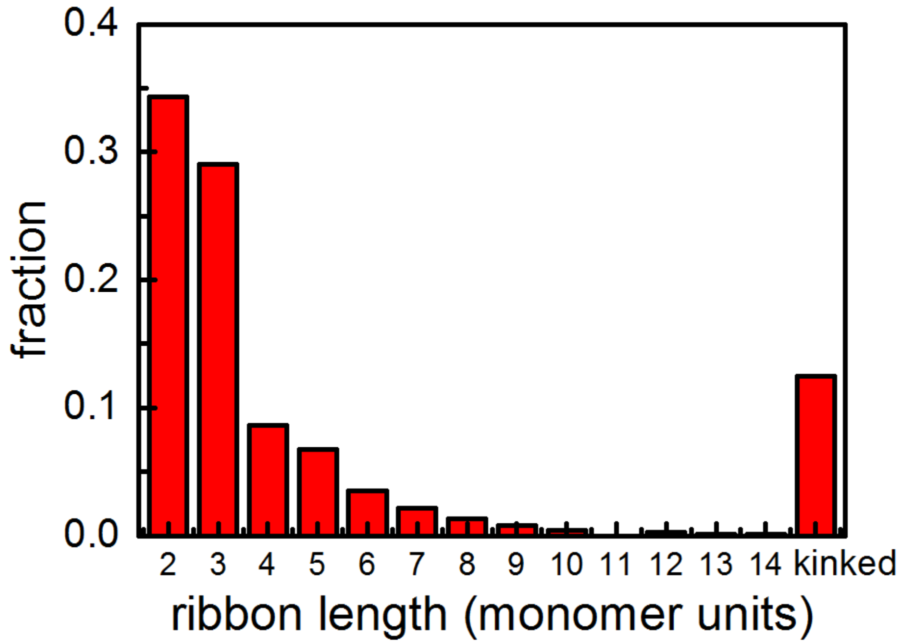

Supplementary Figure 1 | Ribbon length statistics. Distribution of the ribbon lengths and the fraction of kinked ribbons for the preparation conditions of the present study. In total 1350 ribbons were included in the analysis.

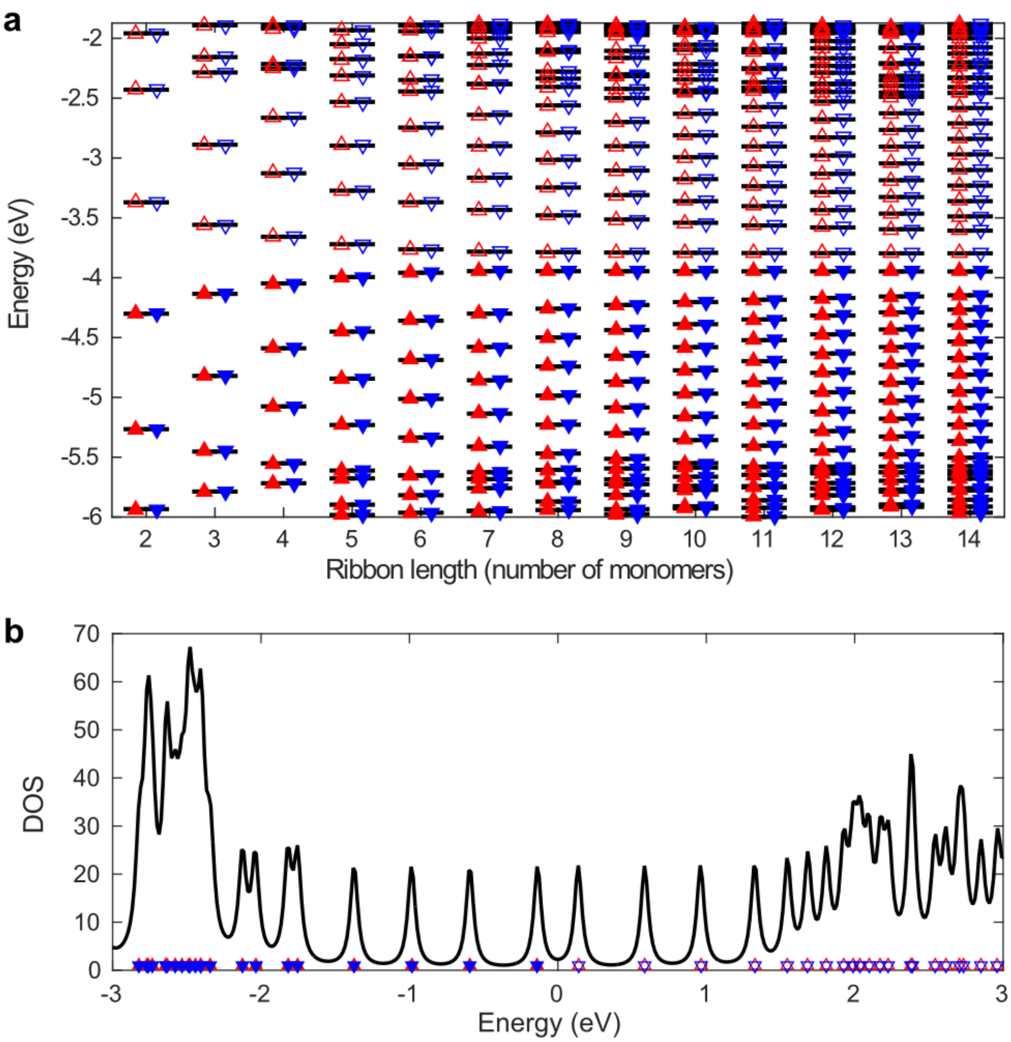

Supplementary Figure 2 | Calculated orbitals as a function of the ribbon length. (**a**) Kohn-Sham energies of the molecular orbitals for the different ribbon lengths. Filled (empty) symbols correspond to occupied (unoccupied) states. (**b**) Calculated density of states (DOS) for an isolated five-monomer ribbon with a Lorentzian broadening of 30 meV. At high bias (above 1.5 V) many close-lying levels contribute to the total DOS.

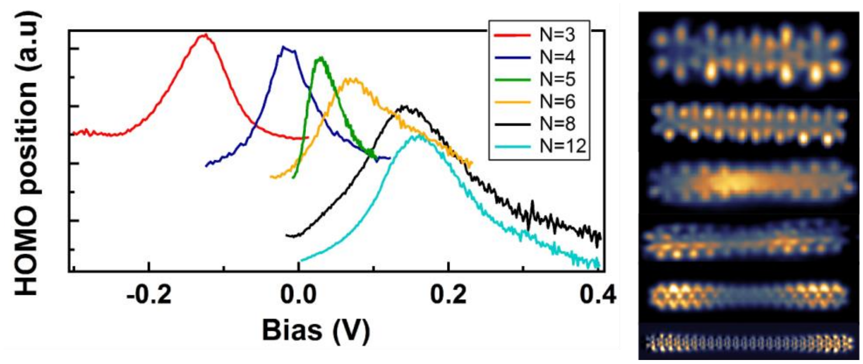

Supplementary Figure 3 | HOMO position as a function of the ribbon length. In shorter ribbons, the highest occupied molecular orbital (HOMO) is located at negative bias. When the ribbon length increases, the HOMO shifts towards positive bias and crosses the Fermi level between four- and five-monomer long ribbons. This means that the HOMO becomes unoccupied and the longer ribbons are charged on Au(111). Left: d*I*/d*V* spectra over the HOMO resonance for different ribbon lengths (*N*=3-6, 8, and 12). Right: Corresponding spatial d*I*/d*V* maps of the HOMO state.

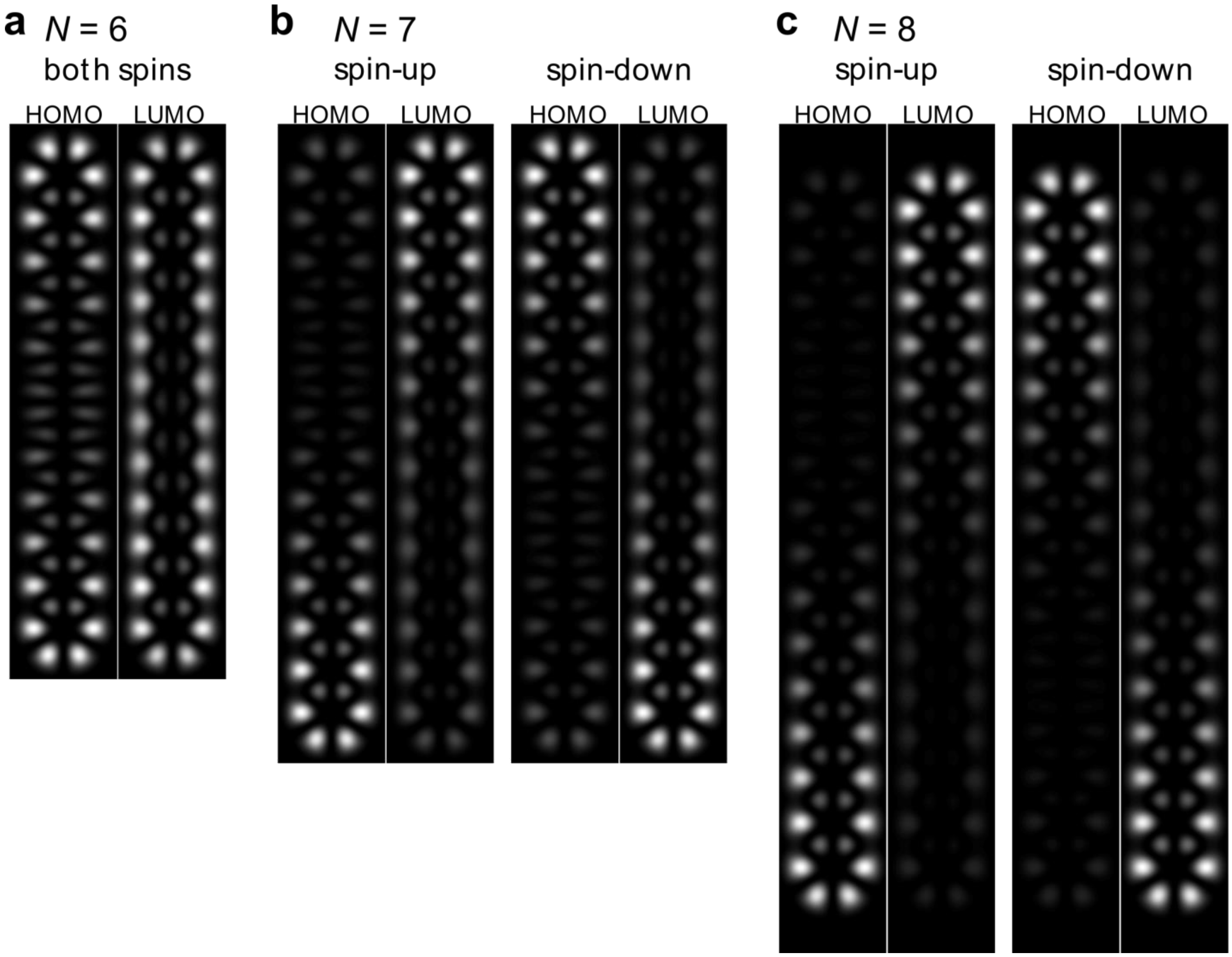

Supplementary Figure 4 | Transition to a spin-polarized ground state with increasing ribbon length. a-c, Simulated constant height d*I*/d*V* maps for GNRs of 6 (**a**), 7 (**b**), and 8 (**c**) monomer units, where the LDOS of each spin channel is shown separately. The maps for the HOMO and LUMO states are shown in each case.

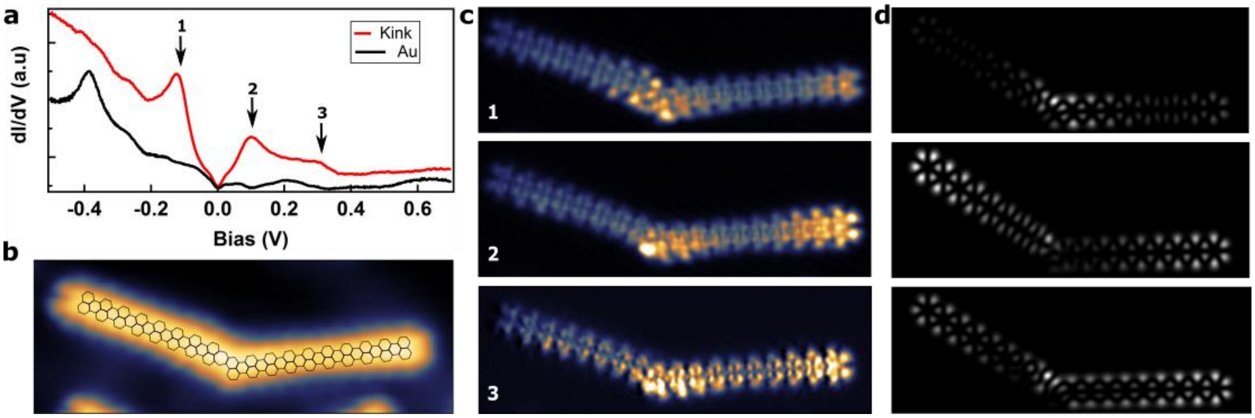

Supplementary Figure 5 | Kinked ribbon with a heptagon connection between the straight segments. (**a**) d*I*/d*V* spectrum acquired at the kink. (**b**) STM topography with model overlaid shows the connection on the kink by a heptagon. (**c**) Constant height d*I*/d*V* map performed at energies labelled by arrows in **a**. (**d**) Corresponding simulated STS maps.

Supplementary Methods

Synthesis of molecular precursor dibromoperylene $C_{20}H_{10}BR_2$ (DBP). Perylene was purchased from TCI. The reagent was reported to be >98.0 % pure, and was used in its initial form. Bromine was stated to be >99.5 % pure, supplied by Riedel-de Haën and used without any further purification. The chemicals used in the work-up processes were stock solutions prepared using commercially available precursors. NMR spectra were recorded on Bruker Avance 400 spectrometer. The spectra were calibrated to CDCl3 (1H: δ 7.27 ppm, 13C: δ 77.0). HRMS spectra were recorded on Waters Micromass LCT Premier (ESI / TOF) mass spectrometer.

A solution of bromine (0.450 ml, 8.74 mmol) in glacial acetic acid (3 mL) was added over 1 h to a solution of perylene (1.060 g, 4.20 mmol) in glacial acetic acid (30 mL) at 40 °C. The reaction was stirred at 50 °C for 5 h. After the mixture was cooled to room temperature the solid product was collected by filtration, washed with water (20 ml), saturated aqueous Na_2CO_3 (40 ml), saturated aqueous NH_4Cl (40 ml) and again with water (40 ml). This resulted in a light brown product with a strong darkish tint. The crude product was purified by recrystallization from toluene to give bright ochre crystalline solid (1.553 g, 90%) as 1:1 mixture of 3,9- and 3,10-dibromoperylene. m.p. 226-228 °C. ^{1}H-NMR (400 MHz, $CDCl_3$): δ 8.27 (dd, *J* = 7.6, 0.7 Hz, 2H), 8.22 (dd, *J* = 7.6, 0.7 Hz, 2H), 8.15 – 8.12 (m, 2 × 2H), 8.02 (d, *J* = 8.1 Hz, 2H), 7.99 (d, *J* = 8.1 Hz, 2H), 7.79 (d, *J* = 8.1 Hz, 2H), 7.78 (d, *J* = 8.1 Hz, 2H), 7.63 – 7.58 (m, 2 × 2H) ^{13}C NMR (100 Mhz, $CDCl_3$) 133.0, 131.1, 131.0, 130.8, 130.7, 130.5, 129.4, 127.9, 127.8, 127.5, 127.4, 123.0, 121.6, 121.3, 121.0, 120.8 HRMS exact mass calculated for $C_{20}H_{10}$ ^{79}Br^{79}Br $[M]^{+\bullet}$: 407.9149, found: 407.9156.

Computational details. The first principles numerical simulations are performed using DFT as implemented in the FHI-aims package[1]. It is an all-electron code that uses local basis functions, and it is therefore an optimal choice for simulating the finite graphene nanoribbons to a good accuracy. We use the default *tight* basis sets for each atom, and the Perdew-Burke-Ernzerhof (PBE) exchange-correlation energy functional[2]. In principle, one could use more sophisticated hybrid functionals and post-DFT methods to treat the electron interactions more accurately, but as the gold substrate screens the interactions, and the substrate is not explicitly included in the simulations, we find that the PBE functional actually shows a good agreement with the measurements.

The nanoribbons are passivated by hydrogen atoms at the edges. The atom positions are relaxed until the forces acting on atoms are less than 10^{-3} eV/Å. In all calculations, the total energy is required to converge to 10^{-6} eV in the self-consistent cycle. The zigzag-edges are initially either with antiferromagnetic or ferromagnetic spin moments, and the one that is lower in energy is chosen as the ground state solution of the spin-polarized calculation.

The DFT calculations show that the ground state solution acquires local spin moments if the ribbons are 7 monomers long or longer. We have reached the same conclusion also using the default *light* basis sets together with the B3LYP exchange-correlation energy functional, which takes some non-local exchange into account as well.

The spin-polarization manifests as the HOMO and LUMO states becoming localized to the zigzag edges, and the two edges having opposite spin moments described as antiferromagnetic (AFM) ordering between the edges. The transition from a trivial spin solution to the AFM spin solution is shown in Supplementary Figure 5, where the constant-height d*I*/d*V* maps, or LDOS, is presented for each spin channel individually for the HOMO and LUMO states.

There are minor differences between the magnetic edge states compared to wider finite armchair nanoribbons[3,4]. Since the 5-carbon-wide ribbons are so narrow, the edge state extends slightly further to the bulk. Moreover, the wave functions near half filling are in principle odd waves in the transverse direction, and thus there is no weight in the states at the central carbon atoms in the longitudinal axis along the ribbon. This also shows as dark (no density) in the simulated d*I*/d*V* maps. These properties already hint why the narrow ribbons that are shorter than 7 monomers cannot support the edge states. We also find that charging the ribbons results either in trivial spin solutions, or at least not the AFM edge states.

The constant-height d*I*/d*V* maps are simulated using the Tersoff-Hamann model for s-type tip symmetry[5]. Namely, it is proportional to the local density of states (LDOS) as

$$\frac{\mathrm{d}I(\vec{r},V)}{\mathrm{d}V} \propto \sum_n |\psi_n(\vec{r})|^2 \delta_\eta((E_n - E_F) - eV) \qquad (1)$$

where ψ_n and E_n are the Kohn-Sham eigenstates and the corresponding eigenenergies, and E_F is the Fermi energy. The delta function is broadened using a Lorentzian function

$$\delta_\eta(E) = \frac{1}{\pi}\frac{\eta}{E^2 + \eta^2} \qquad (2)$$

where we use the broadening parameter η = 30 meV. The d*I*/d*V* maps are evaluated with the tip placed 3.5 Å above the nanoribbon plane. The tip height is taken rather small on purpose, because the basis functions are more accurately described closer to the atoms, where their effect on the total energy is the most crucial.

Supplementary References

1. Blum, V. *et al.* Ab initio molecular simulations with numeric atom-centered orbitals. *Comp. Phys. Commun.* **180**, 2175-2196, (2009).
2. Perdew, J. P., Burke, K. & Ernzerhof, M. Generalized gradient approximation made simple. *Phys. Rev. Lett.* **77**, 3865-3868, (1996).
3. Ijäs, M. *et al.* Electronic states in finite graphene nanoribbons: Effect of charging and defects. *Phys. Rev. B* **88**, 075429, (2013).
4. Golor, M., Koop, C., Lang, T. C., Wessel, S. & Schmidt, M. J. Magnetic correlations in short and narrow graphene armchair nanoribbons. *Phys. Rev. Lett.* **111**, 085504, (2013).
5. Tersoff, J. & Hamann, D. R. Theory and application for the scanning tunneling microscope. *Phys. Rev. Lett.* **50**, 1998, (1983).
